# Supplementary material for: Self-Reported Long COVID and Its Impact on COVID-19-Related Worries and Behaviors After Lifting the COVID-19 Restrictions in China
Source: Healthcare (Basel). 2025 Jan 29;13(3):262. doi: 10.3390/healthcare13030262 (PMC11817031; doi:10.3390/healthcare13030262)
Supplement: Supplementary file 1 [file healthcare-13-00262-s001.zip › healthcare-3421097-supplementary.pdf]

**Table S1: Background characteristics of participants by survey time.**

| Background variables | Survey time, (n, %) 2023.02.07 (n=703) | 2023.03.09 (n=827) | *P* |
|---|---|---|---|
| **Socio-demographic characteristics** | | | |
| Sex | | | 0.885 |
| Male | 392 (55.8) | 457 (55.3) | |
| Female | 311 (44.2) | 370 (44.7) | |
| Age groups (years) | | | **0.005** |
| 18–30 | 257 (36.6) | 342 (41.4) | |
| 31–40 | 193 (27.5) | 248 (30.0) | |
| 41–50 | 127 (18.1) | 142 (17.2) | |
| 51–60 | 80 (11.4) | 65 (7.86) | |
| >60 | 46 (6.54) | 30 (3.63) | |
| Education level | | | 1.000 |
| Below college | 183 (26.0) | 216 (26.1) | |
| College or above | 520 (74.0) | 611 (73.9) | |
| Occupation | | | 0.108 |
| Business/Service staff | 159 (22.6) | 215 (26.0) | |
| Administrative officials | 107 (15.2) | 110 (13.3) | |
| Technical practitioners | 166 (23.6) | 226 (27.3) | |
| Production/Transportation staff or others | 105 (14.9) | 106 (12.8) | |
| Retired/Unemployed | 105 (14.9) | 97 (11.7) | |
| Students | 61 (8.68) | 73 (8.83) | |
| Marital status | | | 0.322 |
| Unmarried | 289 (41.1) | 366 (44.3) | |
| Married | 374 (53.2) | 424 (51.3) | |
| Divorced/Widowed or others | 40 (5.69) | 37 (4.47) | |
| Income level (USD) | | | 0.989 |
| No income | 98 (13.9) | 115 (13.9) | |
| <685 | 198 (28.2) | 228 (27.6) | |
| 685-1371 | 240 (34.1) | 282 (34.1) | |
| >1371 | 167 (23.8) | 202 (24.4) | |
| Duration living in Guangzhou | | | 0.402 |
| >6 months | 673 (95.7) | 783 (94.7) | |
| ≤6 months | 30 (4.27) | 44 (5.32) | |
| **COVID-19 infection-related characteristics** | | | |
| Had chronic diseases | | | 0.357 |
| No | 597 (84.9) | 717 (86.7) | |
| Yes | 106 (15.1) | 110 (13.3) | |
| Time of last COVID-19 infection | | | **0.033** |
| Dec, 2022 | 580 (82.5) | 640 (77.4) | |
| Before Dec, 2022 | 91 (12.9) | 130 (15.7) | |
| After Dec, 2022 | 32 (4.55) | 57 (6.89) | |
| Testing method of COVID-19 | | | 0.602 |

| | | |
|---|---|---|
| RAT[a] | 368 (52.3) | 430 (52.0) |
| NAAT[b] | 107 (15.2) | 128 (15.5) |
| Both RAT[a] and NAAT[b] | 93 (13.2) | 126 (15.2) |
| Symptom-based assessment | 135 (19.2) | 143 (17.3) |

[a]: RAT: rapid antigen test; [b]: NAAT: nucleic acid amplification test

**Table S2:** Prevalence of long COVID symptoms by socio-demographic characteristics (%).

| | Cough | Fatigue | Dyspnea | Palpitations | Insomnia | Cognitive impairment | Depression /anxiety | Dizziness | Chest pain | Headache | Joint pain | Tinnitus/earache | Nausea | Diarrhea | Rash |
|---|---|---|---|---|---|---|---|---|---|---|---|---|---|---|---|
| ALL | 60.7 | 47.6 | 34.5 | 26.2 | 25.1 | 24.0 | 23.6 | 18.5 | 16.4 | 14.9 | 11.3 | 10.5 | 10.2 | 9.1 | 6.6 |
| Sex | | | | | | | | | | | | | | | |
| Male | 9.8 | 6.9 | 5.1 | 3.2 | 3.1 | 3.9 | 3.8 | 1.9 | 2.0 | 2.6 | 1.8 | 1.9 | 1.6 | 1.5 | 0.9 |
| Female | 12.3 | 10.6 | 7.6 | 6.6 | 6.3 | 4.8 | 4.8 | 5.1 | 4.1 | 2.8 | 2.3 | 1.9 | 2.1 | 1.8 | 1.5 |
| Age groups (years) | | | | | | | | | | | | | | | |
| 18–30 | 10.7 | 8.5 | 7.0 | 4.7 | 4.2 | 3.7 | 5.3 | 3.8 | 3.0 | 3.8 | 1.8 | 1.8 | 2.3 | 2.2 | 10.7 |
| 31–40 | 12.9 | 10.2 | 6.6 | 6.1 | 5.0 | 5.2 | 4.3 | 3.2 | 3.4 | 2.5 | 1.6 | 2.3 | 2.5 | 2.3 | 12.9 |
| 41–50 | 11.2 | 8.9 | 6.7 | 3.4 | 3.7 | 6.0 | 3.0 | 2.2 | 2.6 | 0.7 | 1.9 | 1.9 | 0.4 | 0.7 | 11.2 |
| 51–60 | 8.3 | 3.5 | 1.4 | 2.8 | 5.5 | 2.1 | 2.8 | 3.5 | 1.4 | 2.1 | 3.5 | 0.7 | 1.4 | 0 | 8.3 |
| >60 | 5.3 | 7.9 | 5.3 | 5.3 | 5.3 | 2.6 | 2.6 | 4.0 | 4.0 | 2.6 | 4 | 2.6 | 0 | 0 | 5.3 |
| Education level | | | | | | | | | | | | | | | |
| Below college | 9.0 | 5.8 | 3.8 | 3.0 | 3.5 | 3.0 | 4.8 | 2.0 | 3.5 | 3.0 | 4.0 | 2.0 | 1.0 | 2 | 1.3 |
| College or above | 11.6 | 9.5 | 7.1 | 5.3 | 4.9 | 4.8 | 4.1 | 3.8 | 2.7 | 2.6 | 1.3 | 1.9 | 2.1 | 1.5 | 1.1 |
| Occupation | | | | | | | | | | | | | | | |
| Business/Service staff | 10.2 | 8.8 | 7.5 | 4.8 | 4.8 | 5.3 | 5.3 | 4 | 2.9 | 2.9 | 2.4 | 2.1 | 3.5 | 2.1 | 0.5 |
| Administrative officials | 9.7 | 6.5 | 4.1 | 3.7 | 1.8 | 3.2 | 2.8 | 0.9 | 2.3 | 0.9 | 0.5 | 1.8 | 0.9 | 1.4 | 1.4 |
| Technical practitioners | 13.3 | 11.2 | 6.4 | 4.6 | 5.9 | 4.1 | 3.3 | 3.3 | 2.0 | 2.6 | 1.5 | 0.8 | 1.5 | 0.5 | 1.3 |
| Production/Transportation staff or others | 10.4 | 6.2 | 5.2 | 4.3 | 3.8 | 5.7 | 3.8 | 2.8 | 5.2 | 2.8 | 1.4 | 3.3 | 1.9 | 3.3 | 1.9 |
| Retired/Unemployed | 9.9 | 5.9 | 5.4 | 5.4 | 5.0 | 3.5 | 4.5 | 3.5 | 2.5 | 2.5 | 3.5 | 1.5 | 0 | 0 | 1.5 |
| Students | 10.4 | 11.2 | 8.2 | 6.0 | 4.5 | 3.0 | 6.7 | 6.0 | 3.7 | 5.2 | 3.7 | 3 | 2.2 | 3.7 | 0.7 |
| Marital status | | | | | | | | | | | | | | | |
| Unmarried | 11.5 | 8.2 | 7.2 | 5.0 | 4.7 | 3.7 | 5.2 | 3.4 | 3.4 | 3.5 | 1.7 | 1.8 | 2.7 | 2.3 | 0.8 |
| Married | 10.2 | 8.5 | 5.5 | 4.4 | 4.3 | 4.5 | 3.3 | 3.3 | 2.5 | 1.9 | 1.8 | 1.8 | 1.0 | 1.0 | 1.6 |
| Divorced/Widowed or others | 14.3 | 11.7 | 5.2 | 5.2 | 5.2 | 7.8 | 6.5 | 3.9 | 3.9 | 3.9 | 7.8 | 3.9 | 2.6 | 2.6 | 0 |
| Income level (USD) | | | | | | | | | | | | | | | |
| No income | 8.5 | 6.1 | 5.6 | 5.6 | 3.3 | 1.9 | 4.7 | 3.3 | 3.3 | 3.3 | 1.9 | 2.3 | 0.5 | 0.9 | 0 |
| <685 | 10.3 | 9.9 | 6.8 | 4.9 | 5.2 | 6.3 | 6.6 | 3.3 | 3.8 | 3.3 | 3.3 | 3.1 | 2.3 | 2.6 | 1.9 |
| 685-1371 | 11.5 | 7.5 | 5.6 | 3.6 | 3.1 | 2.7 | 2.3 | 3.1 | 2.5 | 1.9 | 1.1 | 1 | 2.1 | 1.1 | 1.1 |
| >1371 | 12.2 | 10.0 | 6.8 | 5.4 | 6.5 | 5.7 | 4.1 | 3.8 | 2.4 | 2.7 | 1.9 | 1.6 | 1.6 | 1.6 | 1.1 |
| Duration living in Guangzhou | | | | | | | | | | | | | | | |
| >6 months | 9.5 | 8.1 | 5.4 | 4.1 | 5.4 | 4.1 | 2.7 | 2.7 | 1.4 | 2.7 | 0 | 0 | 0 | 1.4 | 1.4 |
| ≤6 months | 11.0 | 8.6 | 6.2 | 4.7 | 4.5 | 4.3 | 4.3 | 3.4 | 3.0 | 2.7 | 2.1 | 2.0 | 1.9 | 1.6 | 1.2 |

**Table S3:** Correlations between long COVID symptoms and COVID-19 related worries ($\times 10^3$).

| | Worry about reinfection | Worry about daily life affected by the pandemic | Worry about surging COVID-19 cases in Guangzhou | Worry about surging COVID-19 deaths in Guangzhou | Worry about healthcare system capacity |
|---|---|---|---|---|---|
| Cough | $15.03^{***}$ | $15.81^{***}$ | $3.51^{*}$ | $5.73^{**}$ | $7.88^{***}$ |
| Fatigue | $21.18^{***}$ | $25.70^{***}$ | $12.91^{***}$ | $15.90^{***}$ | $21.37^{***}$ |
| Dyspnea | $24.47^{***}$ | $25.71^{***}$ | $13.57^{***}$ | $12.81^{***}$ | $14.71^{***}$ |
| Palpitations | $14.76^{***}$ | $12.30^{***}$ | $6.56^{**}$ | $6.13^{**}$ | $6.74^{**}$ |
| Insomnia | $15.49^{***}$ | $16.41^{***}$ | $5.38^{**}$ | $13.01^{***}$ | $8.61^{***}$ |
| Cognitive impairment | $23.85^{***}$ | $23.27^{***}$ | $10.81^{***}$ | $14.20^{***}$ | $10.81^{***}$ |
| Depression/anxiety | $24.96^{***}$ | $24.53^{***}$ | $11.66^{***}$ | $12.12^{***}$ | $16.20^{***}$ |
| Dizziness | $5.04^{**}$ | $5.79^{**}$ | 0.40 | 1.09 | $2.81^{*}$ |
| Chest pain | $2.73^{*}$ | $3.06^{*}$ | 0.85 | 2.42 | $3.04^{*}$ |
| Headache | $4.45^{**}$ | $4.16^{*}$ | 0.94 | 1.80 | $4.22^{*}$ |
| Joint pain | $2.79^{*}$ | 1.04 | 0.06 | 0.74 | 1.96 |
| Tinnitus/earache | $6.69^{**}$ | $5.03^{**}$ | 1.36 | $4.36^{**}$ | $3.01^{*}$ |
| Nausea | $5.87^{**}$ | $4.46^{**}$ | 1.07 | $3.84^{*}$ | 1.68 |
| Diarrhea | $8.56^{***}$ | $5.52^{**}$ | $3.66^{*}$ | $6.41^{**}$ | $4.67^{**}$ |
| Rash | 0.70 | 0.82 | 0.85 | 0.61 | 2.30 |

$^{*}$, $p<0.05$. $^{**}$, $p<0.01$. $^{***}$, $p<0.001$

**Table S4:** Univariate *p* values between COVID-19 related worries and background variables.

| | Worry about reinfection | Worry about daily life affected by the pandemic | Worry about surging COVID-19 cases in Guangzhou | Worry about surging COVID-19 deaths in Guangzhou | Worry about healthcare system capacity | Total scores of COVID-19 related worries |
|---|---|---|---|---|---|---|
| | *p value*[a] | *p value*[a] | *p value*[a] | *p value*[a] | *p value*[a] | *p value*[b] |
| **Socio-demographic characteristics** | | | | | | |
| Sex (ref=male) | 0.287 | 0.147 | **<0.001** | **<0.001** | **<0.001** | |
| Female | | | | | | **<0.001** |
| Age groups (ref=18-30 years) | 0.548 | 0.330 | **0.025** | **0.076** | **0.017** | |
| 31-40 | | | | | | 0.218 |
| 41-50 | | | | | | **0.053** |
| 51-60 | | | | | | **0.028** |
| >60 | | | | | | 0.780 |
| Education level (ref=below college) | **0.047** | **0.008** | 0.649 | 0.102 | 0.299 | |
| College or above | | | | | | 0.281 |
| Occupation (ref= business/Service staff | 0.110 | **0.007** | **0.041** | **0.061** | 0.577 | |
| Administrative officials | | | | | | 0.235 |
| Technical practitioners | | | | | | 0.566 |
| Production/Transportation staff or others | | | | | | 0.629 |
| Retired/unemployed | | | | | | 0.176 |
| Students | | | | | | 0.198 |
| Marital status (ref=unmarried) | 0.593 | **0.073** | 0.365 | 0.671 | 0.936 | |
| Married | | | | | | 0.816 |
| Divorced/Widowed or others | | | | | | 0.607 |
| Income level (ref=no income) | **0.022** | **0.001** | **0.009** | **<0.001** | **0.006** | |
| <685 USD | | | | | | 0.225 |
| 685 USD-1371 USD | | | | | | 0.981 |
| >1371 USD | | | | | | **0.018** |
| Duration living in Guangzhou (ref=>6 months) | 0.227 | 0.150 | 0.491 | 0.134 | 0.177 | |
| ≤6 months | | | | | | **0.089** |
| **COVID-19 infection-related characteristics** | | | | | | |
| Had chronic diseases (ref=no) | **0.011** | **0.006** | 0.219 | 0.557 | 0.899 | |
| Yes | | | | | | **0.032** |
| Time of last COVID-19 infection (ref=Dec 2022) | 0.877 | 0.850 | 0.785 | 0.763 | 0.461 | |
| Oct 2022-Nov 2022 | | | | | | 0.623 |
| After Dec, 2022 | | | | | | 0.530 |
| Testing method of COVID-19 (ref=RAT) | 0.715 | 0.395 | 0.482 | 0.744 | 0.587 | |
| NAAT | | | | | | 0.904 |
| Both RAT and NAAT | | | | | | 0.958 |
| Symptom-based assessment | | | | | | 0.530 |
| **Survey time** (ref=Feb 2023) | **0.004** | **<0.001** | **<0.001** | **<0.001** | **0.001** | |
| Mar, 2023 | | | | | | **<0.001** |

[a], *p* values obtained from chi-square test. [b], *p* values obtained from linear regression. *p* values less than 0.1 were in bold.

**Table S5:** Univariate *p* values between preventive behaviors and background variables.

| | Wear masks in public | Wash hands immediately upon returning home | Maintain a one-meter distance in line | Avoid public transport | Avoid social gatherings | Total scores of preventive behaviors |
|---|---|---|---|---|---|---|
| | *p value*[a] | *p value*[a] | *p value*[a] | *p value*[a] | *p value*[a] | *p value*[b] |
| **Socio-demographic characteristics** | | | | | | |
| Sex (ref=male) | **<0.001** | **<0.001** | 0.487 | **0.051** | 0.777 | |
| Female | | | | | | **0.067** |
| Age groups (ref=18-30 years) | **<0.001** | **<0.001** | **<0.001** | **<0.001** | **<0.001** | |
| 31-40 | | | | | | **<0.001** |
| 41-50 | | | | | | **<0.001** |
| 51-60 | | | | | | **<0.001** |
| >60 | | | | | | **<0.001** |
| Education level (ref=below college) | 0.818 | 0.991 | **<0.001** | **<0.001** | **<0.001** | |
| College or above | | | | | | **<0.001** |
| Occupation (ref= business/Service staff | **0.005** | **<0.001** | **<0.001** | **0.001** | **0.004** | |
| Administrative officials | | | | | | 0.591 |
| Technical practitioners | | | | | | **0.069** |
| Production/Transportation staff or others | | | | | | 0.706 |
| Retired/unemployed | | | | | | **<0.001** |
| Students | | | | | | **0.001** |
| Marital status (ref=unmarried) | **0.035** | **<0.001** | **<0.001** | **<0.001** | **<0.001** | |
| Married | | | | | | **<0.001** |
| Divorced/Widowed or others | | | | | | **<0.001** |
| Income level (ref=no income) | 0.364 | **<0.001** | **0.029** | **<0.001** | **<0.001** | |
| <685 USD | | | | | | **<0.001** |
| 685 USD-1371 USD | | | | | | 0.139 |
| >1371 USD | | | | | | 0.378 |
| Duration living in Guangzhou (ref=>6 months) | **0.009** | **0.006** | 0.298 | 0.800 | 0.586 | |
| ≤6 months | | | | | | 0.176 |
| **COVID-19 infection-related characteristics** | | | | | | |
| Had chronic diseases (ref=no) | 0.101 | **0.065** | **0.037** | 0.157 | **0.016** | |
| Yes | | | | | | **0.039** |
| Time of last COVID-19 infection (ref=Dec 2022) | 0.165 | **0.068** | 0.641 | 0.742 | 0.866 | |
| Oct, 2022-Nov, 2022 | | | | | | 0.485 |
| After Dec, 2022 | | | | | | 0.324 |
| Testing method of COVID-19 (ref=RAT) | 0.207 | 0.303 | 0.129 | **0.005** | **0.061** | |
| NAAT | | | | | | **0.002** |
| Both RAT and NAAT | | | | | | 0.234 |
| Symptom-based assessment | | | | | | 0.522 |
| **Survey time** (ref=Feb 2023) | **<0.001** | **<0.001** | **<0.001** | 0.558 | **0.047** | |
| Mar, 2023 | | | | | | **<0.001** |

[a], *p* values obtained from chi-square test. [b], *p* values obtained from linear regression. *p* values less than 0.1 were in bold.
